# A Syrian Child with Intussusception Caused by a Diffuse Large B-Cell Lymphoma

**DOI:** 10.1155/2019/9467630

**Published:** 2019-08-14

**Authors:** Rahaf Toutounji, Ahmad Ghazal, Aghyad kudra Danial, Bayan Toutounji, Tayf Toutounji

**Affiliations:** ^1^Department of Dermatology, Aleppo University Hospital, Aleppo, Syria; ^2^Department of Surgery, Aleppo University Hospital, Aleppo, Syria; ^3^Faculty of Medicine, University of Aleppo, Aleppo, Syria

## Abstract

Intussusception is the invagination of a segment of the bowel into an adjacent segment. Here, we describe a middle-eastern case of an 11-year-old boy who presented to our institution with an intussusception caused by lymphoma but with nonspecific symptoms such as acute abdominal pain and a history of weight loss. His abdominal ultrasound and computed tomography (CT) showed ileocolic intussusception with a mass leading it. We resected the mass with the affected bowel and related lymph nodes, and the histological examination showed features of diffuse large B-cell Non-Hodgkin's lymphoma (an uncommon histologic type in the Middle East). Known as intussusception, this urgent condition could distress infants and young children. However, in older children, the presentation is subtler. So, a lead point should be observed and the chance of malignancy should be kept in mind.

## 1. Introduction

Intussusception is the invagination of a segment of the bowel into an adjacent segment.

The incidence of intussusceptions is approximately 50 per 100,000 individuals, and it is lower among children who are above 6 years old [1].

Primary intussusception is the idiopathic one, and when organic lesions trigger the etiology, we call it secondary.

The pathological lesion (examples are Meckel's diverticulum, polyp, and lymphoma) is present in only 10% of pediatric cases [2].

Although benign lesions are more common, malignant lesions such as malignant lymphoma can also lead the intussusception, especially in older children.

Primary gastrointestinal lymphoma represents 1%-4% of all gastrointestinal malignancies [2].

Abdominal ultrasound scan is the diagnostic test of choice due to its high sensitivity (98–100%), specificity (88%), and low cost [3].

CT is the most sensitive tool, especially in the presence of a lead point; its diagnostic accuracy can reach 100%. We can see the “target” configuration as typical findings on CT. It also allows us to define the lead point and to locate it precisely.

Treatment of intussusception can vary according to each individual case.

We are presenting a case of an 11-year-old child who developed lymphoma as the leading point of intussusception.

To the best of our knowledge, it is one of the few cases of diffuse large B-cell Non-Hodgkin's lymphoma to cause an intussusception and the first case from the Middle East to be published.

## 2. Case Report

An 11-year-old male Syrian child presented to the emergency department of our institution with a 3-day history of acute abdominal pain. In the last two months, the boy lost 5 kg and suffered from lack of appetite and fatigue.

The pain was right-lower quadrant (RLQ) in location, cramping in nature, and accompanied by nausea, vomiting, and constipation. His medical history was unremarkable apart from untreated anemia.

On clinical examination, we found a pale child, a distended abdomen, and an intra-abdominal mass with tenderness in the RLQ. Digital rectal examination revealed empty rectum.

Laboratory tests only showed slight shifting to neutrophils with a hemoglobin level of 11 g/dl.

**Figure 1:**
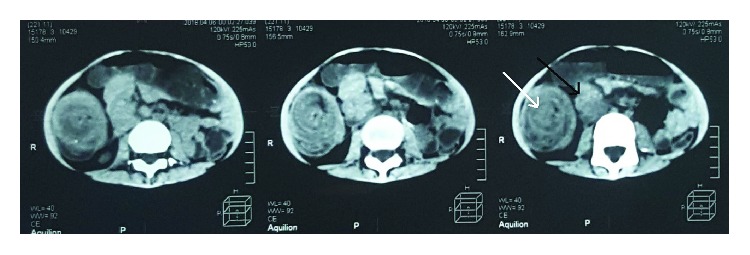
CT showing the “target” configuration (white arrow) and the mass (black arrow).

The abdominal X-ray was unremarkable, and the abdominal ultrasound showed a “target” configuration which appeared in the right lumbar region suggesting an ileocolic intussusception. The CT of the abdomen and pelvis showed dilated small intestinal loops and the “target” configuration again, along with enlarged retroperitoneal and periaortic lymph nodes. It also showed a low-density mass, measuring about 5 cm (Figure 1).

Emergent laparotomy revealed a firm antimesenteric mass, which is located in the terminal ileum and involved in the cecum lumen, acting as the leading point of intussusception.

The intussuscepted segment was congested, the intestinal serosa was necrotized, and the mesenteric lymph nodes were enlarged.

**Figure 2:**
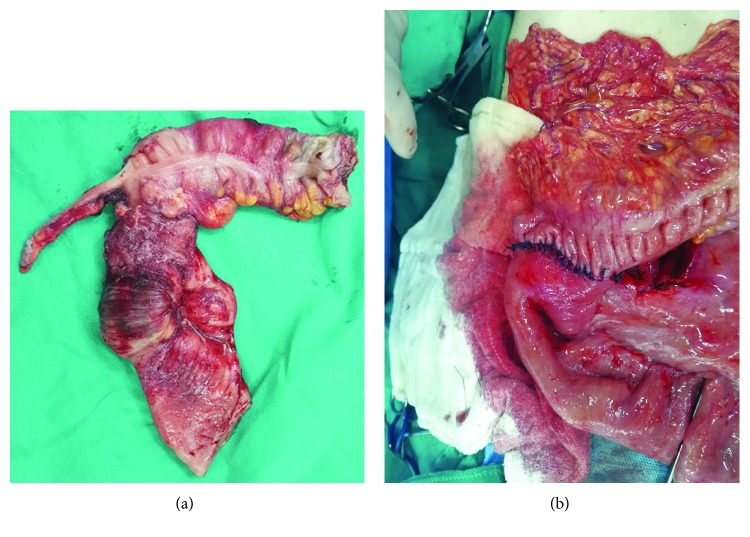
(a) The resected intestines. (b) The anastomosis.

The intussusception was irreducible with proximal compression (Hutchinson's maneuver). Therefore, we resected the affected bowel and did a right hemicolectomy including 6 cm proximal and 8 cm distal margins with the related mesenteric lymph nodes and performed a primary end-to-side, one layer anastomosis (Figures 2(a) and 2(b)).

The histologic examination of the resected specimen confirmed the presence of a large mass (4 × 3 *cm*) with malignant infiltration to peri-intestinal adipose tissue and muscular layer of the ileal wall. These structures showed features of diffuse large B-cell Non-Hodgkin's lymphoma.

## 3. Discussion

Intussusception commonly causes acute intestinal obstruction in pediatric patients. However, a pathologic lead point precipitating the intussusception is less common.

The intussusception is easily diagnosed when occurring under the age of two, and it is characterized by the classic triad of colicky abdominal pain, bloody stools, and a palpable mass.

However, these characteristic findings are observed only in a few patients; moreover, in older children, the presentation of intussusception is subtler and the classic triad of symptoms may not be present. So, the clinical diagnosis alone can be quite challenging as in our case.

The distribution of histologic types of small bowel malignant tumors is largely changing in the world.

Here, in the Middle East and Mediterranean basin, primary small intestinal lymphoma is usually of the immunoproliferative small intestinal disease (IPSID) type, and it is more common in adult patients [4].

However, pathologic examination of our patient's intestines showed features of diffuse large B cell lymphoma, which is a western-type, non-IPSID lymphoma.

Moreover, gastrointestinal lymphoma rarely causes intussusception. In fact, it could add different nonspecific symptoms to the clinical aspect, such as anorexia and weight loss. A high index of suspicion is then required to put the right diagnosis and that is what we did with our case.

We identified the intussusception mass on echo examination, but our concerns about a pathologic point leading the intussusception made the CT our next choice.

As CT showed, our patients' mass and “target” configuration were located in the right lumbar area.

Primary ileocolic intussusception is successfully reducible by conservative reduction with pneumatic or hydrostatic pressure.

In older children and adults, however, most cases of intussusception are secondary to a pathologic lead point as in our case, and such conservative methods are usually not sufficient and time-wasting.

Segmental bowel resection is then necessary and the laparoscopic technique may not be appropriate, considering the very intensive need to resect the leading lesion [5].
